# Sequence analysis and variant identification at the *APOC3* gene locus indicates association of rs5218 with BMI in a sample of Kuwaiti’s

**DOI:** 10.1186/s12944-019-1165-6

**Published:** 2019-12-19

**Authors:** Zainab H. Malalla, Ahmad E. Al-Serri, Huda M. AlAskar, Wafaa Y. Al-Kandari, Suzanne A. Al-Bustan

**Affiliations:** 1 Department of Biological Sciences, Faculty of Science, Kuwait University, Kuwait, Kuwait; 2 Human Genetics Unit, Department of Pathology, Faculty of Medicine, Kuwait University, Hawally, Kuwait

**Keywords:** *APOC3*, Sequence variants, Genetic association, Lipid levels, BMI, Arabs

## Abstract

**Background:**

APOC3 is important in lipid transport and metabolism with limited studies reporting genetic sequence variations in specific ethnic groups. The present study aimed to analyze the full *APOC3* sequence among Kuwaiti Arabs and test the association of selected variants with lipid levels and BMI.

**Methods:**

Variants were identified by Sanger sequencing the entire *APOC3* gene in 100 Kuwaiti Arabs. Variants and their genotypes were fully characterized and used to construct haplotype blocks. Four variants (rs5128, rs2854117, rs2070668, KUAPOC3N3 g.5196 A > G) were selected for testing association with serum lipid levels and BMI in a cohort (*n* = 733).

**Results:**

*APOC3* sequence (4.3 kb) of a Kuwaiti Arab was deposited in Genbank (accession number KJ437193). Forty-two variants including 3 novels were identified including an “A” insertion at genomic positions 116,700,599–116,700,600 (promoter region) and two substitutions in intron 1 at genomic positions 116,700,819 and 116,701,159. Only three variants, (rs5128, rs2854117, and rs2070668) were analyzed for association of which rs5128 showed a trend for association with increased BMI, TG and VLDL levels that was further investigated using multivariate analysis. A significant association of rs5128 with BMI (*p* <  0.05) was observed following a dominant genetic model with increased risk by an OR of 4.022 (CI: 1.13–14.30).

**Conclusion:**

The present study is the first to report sequence analysis of *APOC3* in an Arab ethnic group. This study supports the inclusion of rs5128 as a marker for assessing genetic risk to dyslipidemia and obesity and the inclusion of the novel variant g.5196 A > G for population stratification of Arabs.

## Introduction

Apolipoprotein C3 *(APOC3)* has been implicated as an important candidate gene involved in plasma lipid level variation and other metabolic abnormalities. The *APOC3* gene resides within the *APOA5-APOA4-APOC3-APOA1* multi-gene cluster on human chromosome 11q23 [1]. The *APOC3* gene is 3367 bp comprising 4 exons that encode a 99 amino acid glycoprotein which is synthesized mostly in the liver and to a lesser degree in the intestine, where it undergoes intracellular cleavage of 20-aminoacid-residue signal peptide yielding the mature 79 amino acid APOC3 [2]. APOC3 is a major protein constituent of triglyceride-rich lipoproteins (TRLs) including very low-density lipoprotein (VLDL), and chylomicron (CM) wherein it has a principal role in the regulation of triglyceride rich lipoproteins (TRL) catabolism [3]. APOC3 impairs lipolysis of TRL by inhibiting lipoprotein lipase (LPL) and the hepatic uptake of TRLs by remnant receptors. High circulating concentration of APOC3 was shown to be associated with increased levels of triglycerides (TG) in blood [4, 5] and in metabolic disorders including dyslipidemia [6, 7]. Dyslipidemia covers a broad spectrum of lipid abnormalities including elevated levels of plasma triglyceride (TG) and total cholesterol (TC), an increase in intermediate-density lipoprotein (IDL), presence of small dense low-density lipoprotein (LDL) particles, and a decreased level of high-density lipoprotein (HDL) [8]. Heritability studies revealed a strong genetic component to dyslipidemia, ranging from 0.20 to 0.60 in which these estimates are likely reflecting contributions from numerous gene variants including *APOC3* [9–14].

The important role of APOC3 in lipid transport and metabolism deems it necessary to reveal the full genetic profile of single nucleotide polymorphisms (SNPs) at this locus and how they may correlate to inter-ethnic susceptibility of dyslipidemia and other metabolic abnormalities. Most of the genetic variants studied in the *APOC3* gene are localized in the promoter region including the five common DNA polymorphisms (C-641A, G-630A, T-625 deletion, C-482 T, and T-455C) that show a minor allelic frequency of about 40% in the general population [15–18]. Sequence analysis of the full locus may provide an effective opportunity to assess the full spectrum of variants and how they may influence plasma lipid levels including both common and rare variants. Recently, exome sequencing of the APOCIII gene locus identified rare loss of function mutations associated with lower triglyceride levels in a large cohort [19]. Very few studies have investigated the role of *APOC3* in the genetic predisposition to variation in lipid levels among Arabs [20] and none have reported full sequence analysis of the *APOC3* gene locus in any Arab population (as far as our knowledge) while limited studies reported sequence analysis on Asians [21].

This paper aimed to investigate sequence variation at the *APOC3* gene locus to identify potential variants that may contribute to the variation in lipid levels and specifically the genetic profile of an Arab population. This study was based on the hypothesis that there are genetic variants that vary in their frequency between different human populations so a common SNP in one ethnic population could be rare in another. Moreover, some *APOC3* SNPs could be significantly associated with variation in lipid levels but are dependent on ethnicity. Therefore, the data generated in this study may allow better identification and selection of associated variants with variation in serum lipid levels and/or BMI.

## Methods

### Study population

**Fig. 1:**
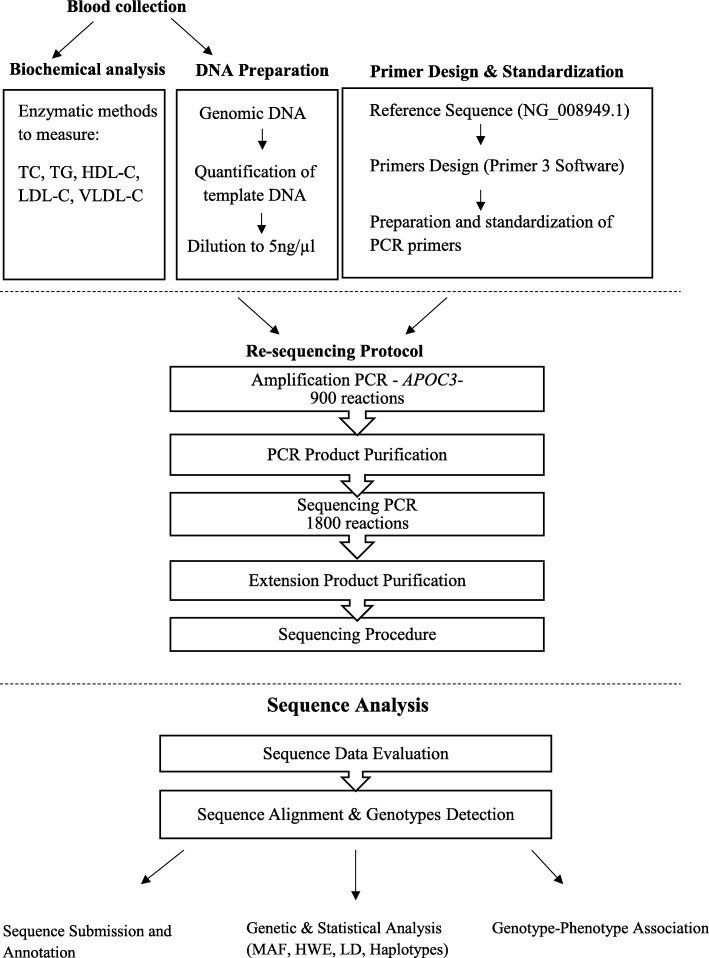
Summary flowchart representing the methodology used for re-sequencing the APOC3 gene locus in this study

This study involved two sample phases. The first was sequencing DNA samples from 100 apparently healthy subjects (50 females and 50 males) with Arabian origin from the Kuwaiti population. The age of the subjects ranged from 18 to 69 with an average age of 30.42 years. The BMI of the subjects ranged from 19 to 30 with an average BMI of 23.946 kg/m. The samples included Kuwait Arabs with normal lipid levels. Each sample had documented phenotypic data including medical and family history of hypertension, hypertriglyceridemia, hypercholesterolemia, diabetes and cardiovascular diseases as well as a pedigree with ethnic background. The pedigree was used to trace the ethnic background of both paternal and maternal lineages back at least four generations. The second phase involved 633 samples of the general the Kuwaiti population for the association of selected variants and validation of the novel variants making the cohort for the genetic association study to 733. Random Kuwaitis were recruited during routine check-up at different governmental hospitals around Kuwait. The inclusion criteria included participants who are Kuwaiti natives with documented ancestry and were above the age of 18 years. The exclusion criteria were those with documented diagnosis of diabetes mellitus type 2, hypertension and heart disease. The study population consisted of 438 females and 295 males. The age of the subjects ranged from 18 to 76. Demographic information, along with detailed personal medical and family history from all the participants (Table 1). The inclusion criteria included participants who are Kuwaiti natives with documented ancestry and were above the age of 18 years. The exclusion criteria were those with documented diagnosis of diabetes mellitus type 2 and/or hypertension. All the participants in this study were devoid of diabetes and/or hypertension. A summary of the procedures followed on the collected blood samples is illustrated in Fig. 1.

**Table 1:** Demographic and clinical features of the Kuwaiti cohort (*n* = 733)

Parameter	
Sex (Males, Females)	40.2 59.8%
Age (year)	32.6 ± 0.52
BMI	27.2 ± 0.29
Cholesterol (mmol/L)	4.7 ± 0.04
Triglyceride (mmol/L)	0.8 ± 0.77
VLDL (mmol/L)	0.3 ± 0.33
HDL (mmol/L)	1.1 ± 0.40
LDL (mmol/L)	3.0 ± 1.10

*BMI* Body mass index, *VLDL* Very low-density lipoprotein, *HDL* High density lipoprotein, *LDL* Low density lipoprotein, *mmol/L* millimoles per liter

### Collection of blood samples and biochemical analysis

Venous blood samples were taken after a 12-h fast. The levels of serum TC, TG, HDL-C, and LDL-C were determined by enzymatic methods with commercially available kits and performed on a UniCel DxC 800 Synchron Clinical System from Beckman Coulter (USA) in the Clinical Chemistry facility at Al-Amiri Hospital (Kuwait).

TC was measured using an enzymatic colorimetric method that breaks it down into water and quinon-imine red dye which is directly proportional to TC concentration. For TG, multienzymatic reaction using glycerol kinase, glycerol-3-phosphate oxidase and peroxidase was performed in a sequence to get a red dye. TG concentration is proportional to the intensity of the color generated and measured photometrically. For HDL-C measurement, a unique detergent was used that is not only able to selectively solubilize cholesterol in HDL but also to inhibit its release from other lipoproteins. Released HDL-C was then determined enzymatically using cholesterol esterase and cholesterol oxidase to produce a color product that could be measured at 560 nm. Friedewald formula was used to calculate the concentration of both LDL-C and VLDL-C (LDL-C = TC – HDL-C – (TG/2.2, VLDL-C = TG/2.2) [22]. The reference values used in this study are those set by Kuwait Ministry of Health where: TC = 3.0–5.17 mmol/L, TG = 0.40–1.7 mmol/L, HDL-C = 0.91–2.5 mmol/L and LDL-C = 1.8–3.2 mmol/L.”

### DNA extraction and *APOC3* sequencing

Total genomic DNA was extracted from whole blood samples, based on the technique described by Miller using proteinase K and salting-out procedures [23]. The 3.4 Kb *APOC3* gene along with the flanking sequences was amplified using nine sets of custom designed overlapping primers (Primer3 Input software version 0.4.0: //Frodo.wi.mit.edu/). The primers and polymerase chain reaction (PCR) conditions are provided in Additional file 1: Table S1. DNA template was first amplified by PCR using Gen Amp® Fast PCR Master Mix in an Applied Biosystem Fast thermal cycler (Version 1.01, Life Technologies, USA) (Additional file 1: Table S2) followed by purification using NucleoSpin® Extract II (Clontech Laboratories, Inc., Version No. PR48598) Kit and formaldehyde denaturation. Sequencing reactions were performed on both DNA strands using BigDye X. Terminator v.1.1 Cycle Sequencing Kits (Additional file 1: Table S2). Sanger bidirectional sequencing was performed using the Gene Analyzer 3130XL (Life Technologies, Applied Biosystems, USA), supported by ABI DNA Sequencing Analysis Software v5.2. The sequences from each pair of reaction were aligned together and checked for sequence accuracy using Clustal W pairwise sequence alignment. Multiple sequence alignment among all samples were compared to the reference sequence (NG_008949.1) in the GenBank database (http://www.ncbi.nlm.nih.gov, NCBI) to identify all the *APOC3* variants among the 100 Kuwaiti Arab samples sequences.

### Validation and association of common and novel SNPs identified

Three common SNPs (MAF >  0.05) identified (rs5128, rs2854117, and rs2070668) were selected for association analysis with variation in lipid levels along with the two novel variants (KUAPOC3N2, and KUAPOC3N3) which were tested for validation among larger cohort (*n* = 733). Allelic discrimination (VIC- and FAM-labeled) using real-time PCR (ABI 7800HT Realtime PCR (GS01/02) was performed for all of the five selected variants. Assay-on-demand of the commercially available TaqMan assays were ordered for the four common variants, while a set of customized primer and probe were used for the two novel variants (Table 2). The reaction was carried according to the instruction of the manufacturer (Applied Biosystem) using TaqmanTM Genotyping Master Mix (Applied Biosystems # 4371355). For quality control, samples were tested on duplicates to estimate genotyping reproducibility; concordance exceeded 99%.

**Table 2:** Selected SNPs and their relevant information. Reported Minor Allele Frequency and predicted consequence were obtained from http://www.ncbi.nlm.nih.gov/snp/, NCBI

SNP	Position (GRCh38.p12)	Consequence	GenomAD reported MAF	Context sequence
rs5128 (C3238G)	chr11:116832924 g.8017G > C	3’UTR Variant	G = 0.16051 (39,468/245886)	AAGTCCACCTGCCTATCCATCCTGC [C/G]AGCTCCTTGGGTCCTGCAATCTCCA
rs2854117 (C-482 T)	chr11:116829426 g.4519 T > C	5’UTR Transcript Variant	C = 0 0.4163 (12,835/30834)	AGGCCTTGCCGGAGCCACTGATGCC [T/C]GGTCTTCTGTGCCTTTACTCCAAAC
rs2070668 (T528G)	chr11:11683043 g.5530 T > G	Intron (1) Variant	G = 0.4314 (13,268/30758)	[T/G]AGCAGGGAGCCGGCCCCTACTCCTT
KUAPOC3N2	chr11:116700819 g.5196 A > G	Intron (1) Variant	N/A	Custom Designed NovelSNP2: Forward: GGTCCTCAGTGCCTGCTG Reverse:TCTAGGGATGAACTGAGCAGACA
KUAPOC3N3	chr11:116701159 g. 5536G > A	Intron (1) Variant	N/A	Custom Designed NovelSNP3: Forward: CCCCCACCCCTCATCATAAC Reverse: CTATGTAGCTTTGGGCAAGTGA

*SNP* Single nucleotide polymorphism, *3′UTR* 3′-untranslated region, *5′UTR* 5′-untranslated region, *N/A* Not available

### Statistical analysis

Genotypic and allelic frequencies were determined by simple gene counting. The chi-square test was used to test the Hardy-Weinberg equilibrium (HWE) within the sample population. Haploview program v4.2 was used to check linkage disequilibrium (LD) between SNPs and construct haplotypes. The possible association of lipid profile with *APOC3* polymorphisms was initially examined with regards to age, gender and BMI using SPSS v21.0. The R software v3.3.1 was used for further analysis utilizing the following packages SNPassoc, psych, genetics, and MASS [24]. Kruskal-Wallis ANOVA test was performed, and the results were reported as mean ± standard error. Log-transformation was applied across all of the lipid profile values (HDL-C, LDL-C, VLDL, and TG) so as to achieve an approximate normal distribution. Additionally, logistic regression model was used to check for any possible association between the studied SNPs and lipid profile parameters. Genetic modeling of the significant variants was performed. A *p*-value of 0.05 was considered statistically significant.

## Results

### *APOC3* sequence analysis

**Fig. 2:**
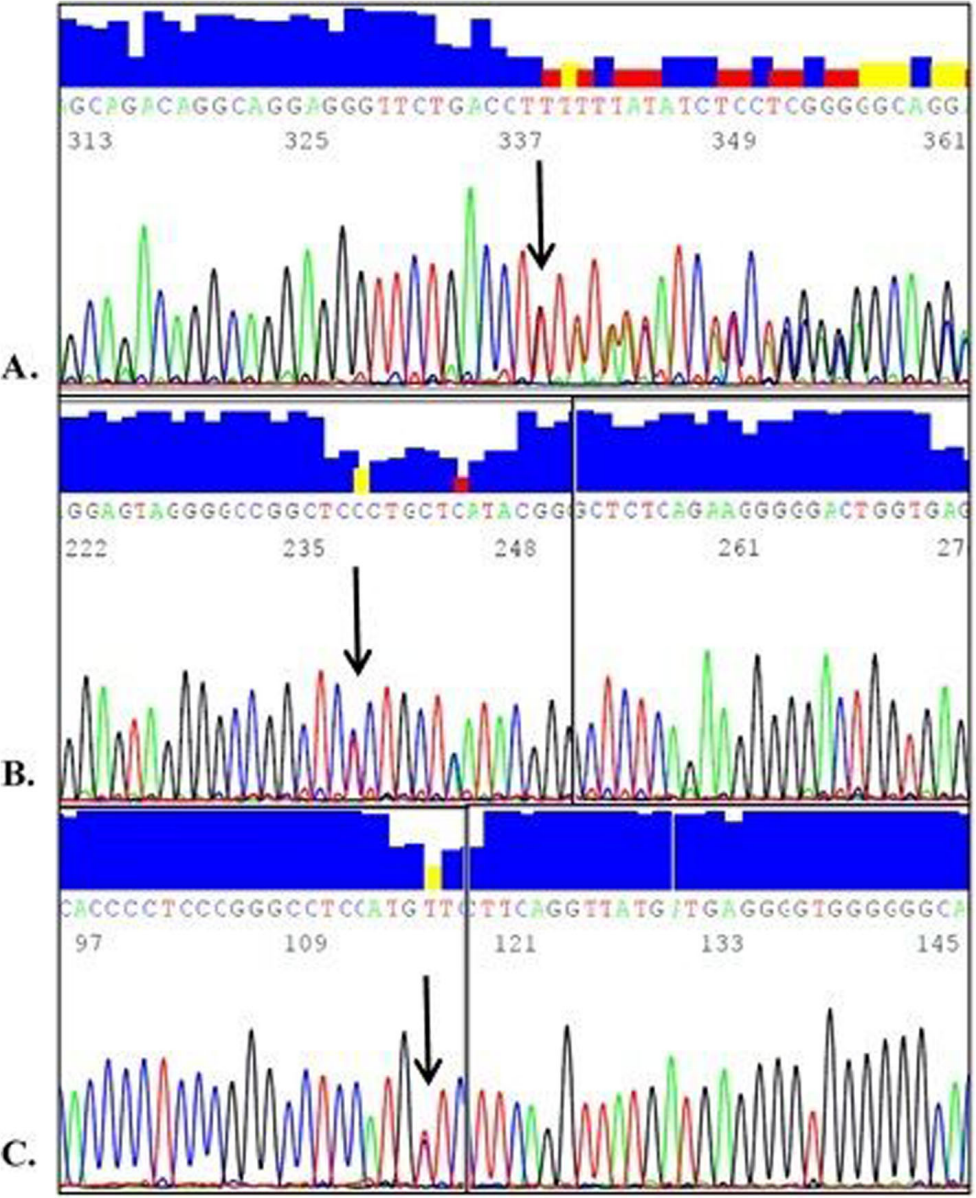
Parts of the APOC3 reverse sequence electropherograms showing the three identified novel SNPs. Each novel SNP is indicated with an arrow on the figure and was confirmed by sequence alignment with the sequence generated by the forward primer. **a** A novel heterozygote (del/A) within the TATA box in the promoter region. **b** A novel heterozygote (A/G) within the first intron. **c** A novel heterozygote (G/A) within the first intron

The full nucleotide sequence of the *4.3* Kb *APOC3* gene locus among 100 Kuwaiti Arabs was analyzed excluding the 224 bp repetitive segment spanning nucleotide positions 2366–2589 due to the technical difficulties generated by the sequence analysis software. The newly defined *APOC3* gene sequence in the Kuwaiti Arab samples was deposited in the NCBI gene bank with accession number (GenBank: KJ437193). Sequence analysis identified 45 different polymorphisms including 42 previously reported SNPs and 3 novel SNPs (Fig. 2).

All three novel variants were found in a heterozygous state. The first novel variant (KUAPOC3N1) is an insertion of one nucleotide (A) within the promoter region (25 bases upstream of the gene) between positions g.4976 and g.4977 relative to the GenBank sequence (NG_008949.1) corresponding to genomic positions 116,700,599 and 116,700,600 in the newly generated sequence (accession number: KJ437193) and was detected in two normolipidemic individuals. The other two novel SNPs were found within the first intron of the *APOC3* locus, each of them in one individual of normal lipid profile. One variant (KUAPOC3N2) resulted from A to G transition at genomic position 116,700,819 on the newly generated *APOC3* sequence (KJ437193) (NG_008949.1 g.5196 A > G). The other variant (KUAPOC3N3) resulted from G to A transition at genomic position 116,701,159 (KJ437193) (NG_008949.1 g. 5536G > A).

**Fig. 3:**
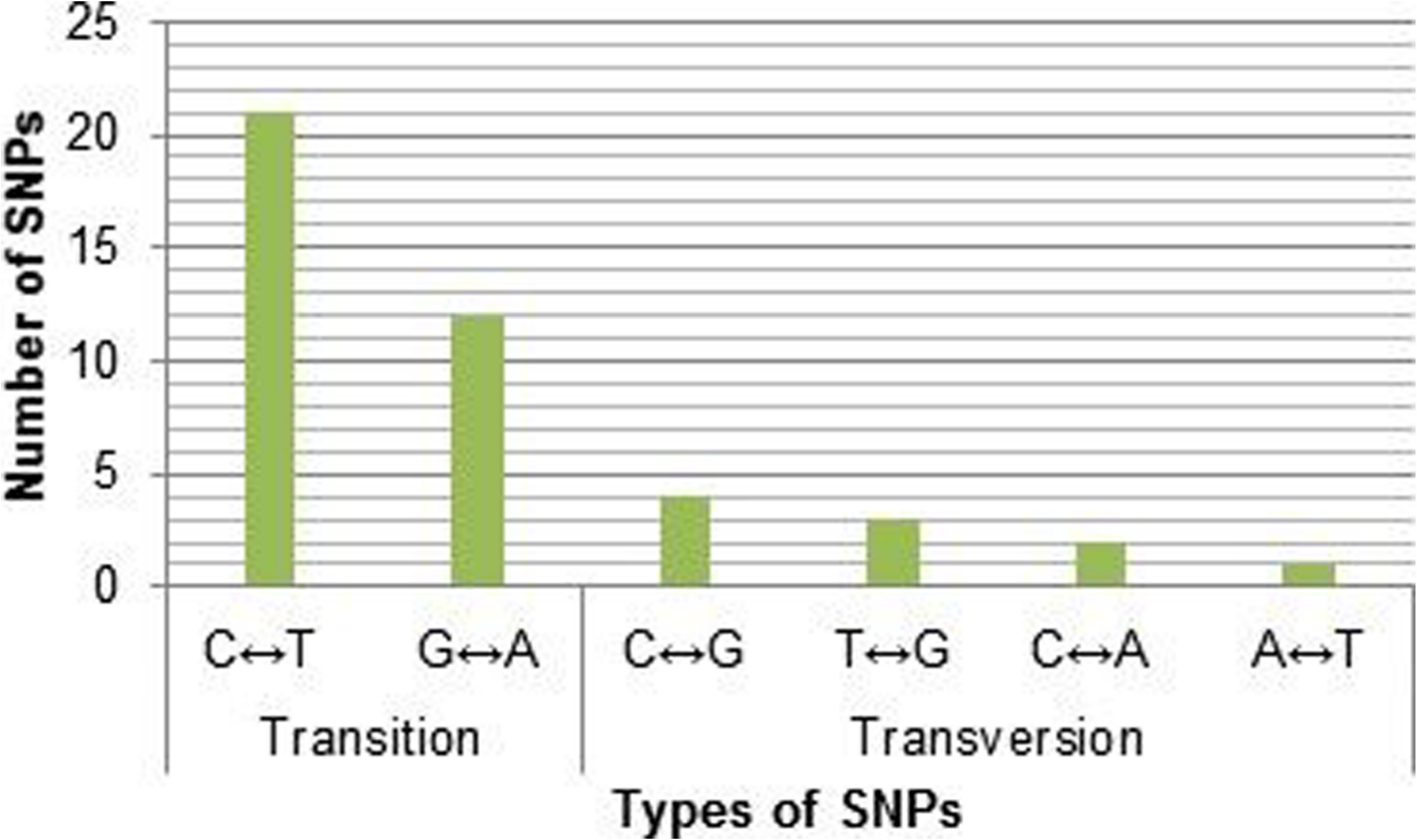
Substitution SNPs (*n* = 43) at the APOC3 gene locus among the analyzed 100 Kuwaiti Arab samples in term of their types

**Fig. 4:**
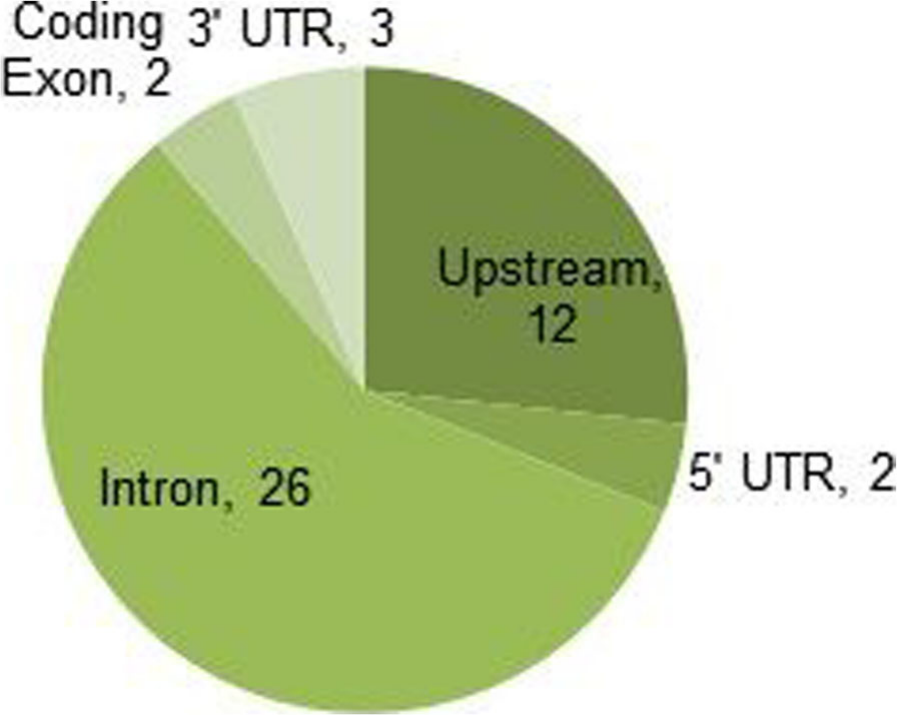
Identified SNPs (*n* = 45) at the APOC3 gene locus among the analyzed Kuwaiti Arab samples (*n* = 100) in term of their location

The remaining 42 variants were mainly SNPs with only 1 InDel. In general, a higher number of transitions type substitutions (*n* = 31) was observed compared to transversions (*n* = 10) (Fig. 3). Considering the individual substitutions, C to T (*n* = 21) was found to be predominant when compared to others G to A (*n* = 9), G to C (*n* = 4), G to T (*n* = 3), C to A (*n* = 2), and T to A (*n* = 1). Most of the identified SNPs were observed in non-coding regions, especially in intronic sequences totaling 26 SNPs (Fig. 4). There were 12 SNPs upstream of the gene, 2 SNPs in the 5′-UTR, and 3 SNPs in the 3′-UTR. Interestingly, there were only 2 SNPs found within the coding exons; the nonsense mutation rs76353203 (R19X) and the synonymous mutation rs4520 in exon 3 and exon 4 respectively.

### SNPs and haplotype analysis

Analysis of the 45 SNPs in this study found 22 SNPs to have a minor allele frequency (MAF) more than 5% (Table 3) while the remaining 23 SNPs showed MAF < 5% (Additional file 1: Table S3). Most of the identified SNPs were found to be in HWE (*p*-value > 0.05) except for 6 SNPs (*p*-value < 0.05). Deviated SNPs included: rs2854117 (*p*-value < 0.001), rs734104 (*p*-value = 0.012), rs5142 (*p*-value = 0.0018), rs5141 (*p*-value = 0.001), rs645901 (*p*-value = 0.013), and rs5128 (*p*-value = 0.006). For all of the 6 SNPs, the homozygous genotypes were over-represented at the expense of heterozygous genotypes.

**Table 3:** Genotypic and allelic frequencies for the 5 selected APOC3 SNPs (*n* = 733) that were genotyped by Realtime PCR

SNPs		Genotypic frequencies (*n* = 733)			Allelic frequencies		*P-value for HWE
		AA	AM	MM			
1	rs5128 (g.8017G > C)	0.06 *n* = 44	0.26 *n* = 190	0.68 *n* = 499	G = 0.19	C = .81	< 0.005
2	rs2854117 (g.4546C > T)	0.40 *n* = 292	0.45 *n* = 334	0.15 *n* = 107	C = 0.63	T = 0.37	< 0.005
3	rs2070668 (g.5530 T > G)	0.18 *n* = 131	0.47 *n* = 344	0.35 *n* = 258	T = 0.41	G = 0.59	> 0.05
4	KUAPOC3N2 (g.5196A > G)	0.9998 *n* = 732	0.0002 *n* = 1	0 *n* = 0	A = 0.9997	G = 0.0003	< 0.005
5	KUAPOC3N3 (g. 5536G > A)	0.984 *n* = 721	0.016 *n* = 12	0 *n* = 0	G = 0.999	A = 0.001	> 0.05

*SNP* Single nucleotide polymorphism, *A* ancestral allele and *M* mutant allele, *HWE* Hardy-Weinberg Equilibrium

*significance value set at *p*<0.05

**Fig. 5:**
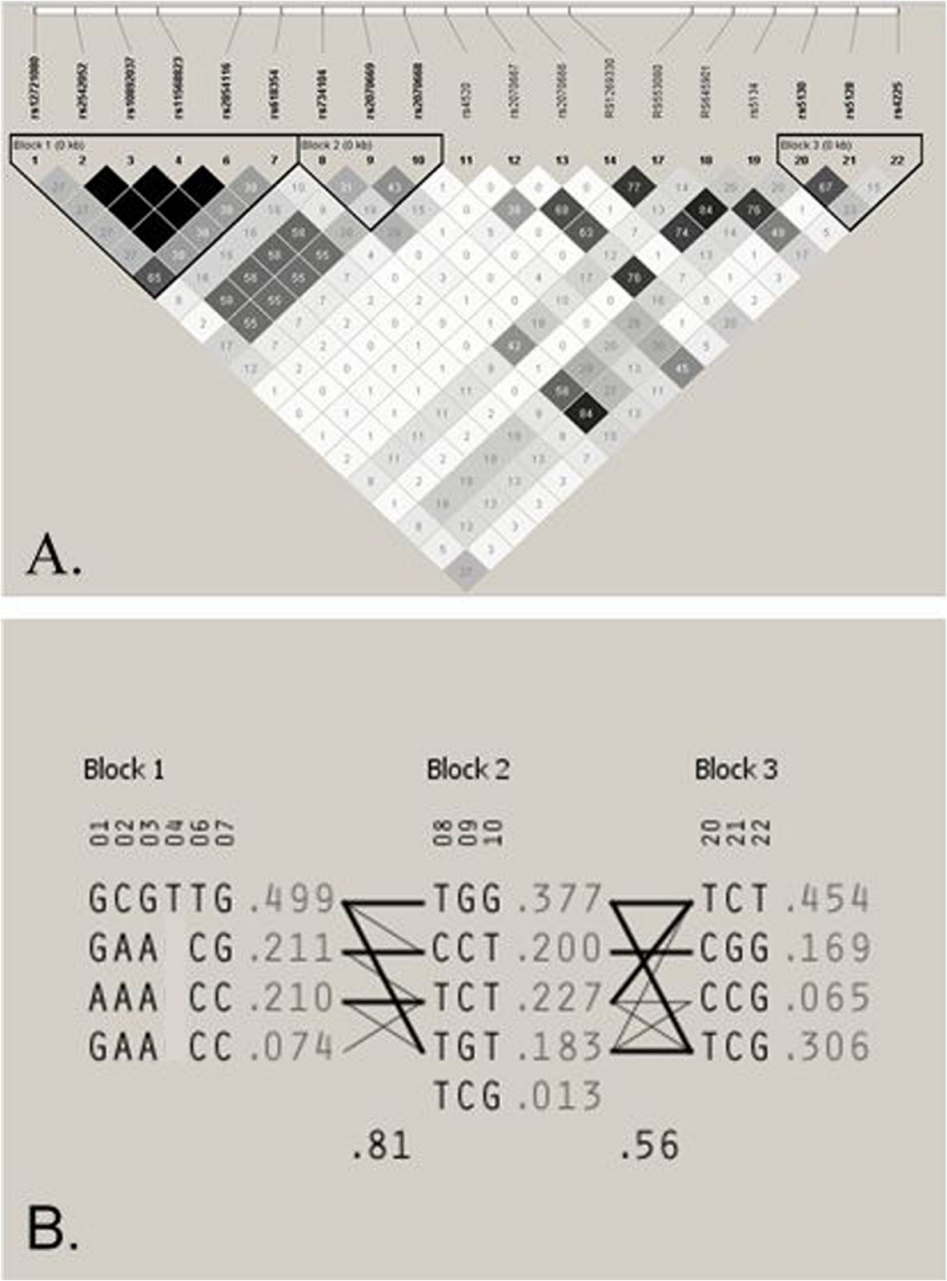
Linkage disequilibrium structure and haplotypic architecture in APOC3. **a** Haploview plot defining haplotype block structure of the *APOC3* gene locus. Haplotype blocks are outlined in bold. Shading indicates strength of linkage disequilibrium between the SNPs as measured by r^2^, which is provided in the intersecting squares. r^2^ is not displayed for squares with *r*^2^ = 1. A diagram of the *APOC3* gene structure is provided over the plot where the first and last SNPs are on the left and right sides of the diagram, respectively. Under the SNP ID numbers are the index numbers, shown in bold, for the SNPs based on the map file. **b** Haplotypes in the haplotype blocks across the *APOC3*. There are three haplotype blocks across the gene. The haplotype frequencies are shown to the right of each haplotype. Only haplotypes having a frequency > 1% are shown. The SNP numbers across the top of the haplotypes correspond to those in the Haploview plot. A multiallelic D′ statistic, which indicates the level of recombination between two blocks, is shown in the crossing area. Connections from one block to the next were shown for haplotypes of > 10% frequency with thick lines and > 1% frequency with thin lines

As variants with low MAF (< 5%) are more prone to statistical error and false findings, only common variants were further analyzed. Haplotype analysis using 22 SNPs resulted in five common (> 5%) haplotypes in three blocks (Fig. 5). The first haplotype block consists of the 5′ promoter SNPs (rs12721080, rs2542052, rs10892037, rs11568823, rs2854116) and rs618354 within the first intron. The second haplotype block includes 3 consecutive polymorphisms within the first intron rs734104, rs2070669, and rs2070668. The third haplotype block includes rs5130 in intron 3 along with both rs5128 and rs4225 within the untranslated region of exon 4. Linkage analysis between SNPs were measured, with results showing four SNPs (rs2542052, rs10892037, rs11568823, and rs2854116) to be in complete LD (*r*^2^ = 1) (Additional file 1: Table S4).

### Validation and association of selected variants with serum lipid levels

Three SNPs (rs2854117, rs2070668, rs5128) were selected for further association analysis as they have been reported to be associated with lipid variation in other populations [21–27]. Genotyping of the three selected variants and the two novels were determined based on the allelic discrimination assay using real-time PCR. The genotypes obtained for the novel variants in the initial sequencing procedure were also observed by real-time PCR (Additional file 2: Figure S1). The allele frequencies in the cohort obtained from real-time PCR (Table 3) were mostly found to be consistent with the frequencies obtained using the sequencing method (*n* = 100) (Additional file 1: Table S3). Deviation from HWE was observed for rs5128, most likely an outcome of excessive homozygous carriers of the mutant wild type C allele in the studied population. The two novel variants were only identified in heterozygous states in < 1% of the cohort (*n* = 733) in which Novel 3 was identified in 10 additional samples while Novel 2 was not identified in any other sample than the originally sequenced sample. Novel 2 also showed deviation from HWE (*p* <  0.005) most likely due to status as a very rare allele and therefore was excluded from further association tests.

Analysis for genetic association of the selected SNPs with serum lipid level and BMI employing Kruskal-Wallis ANOVA after adjustment for sex, age and BMI did not show any significant associations (*p*-value > 0.05) (Table 4). However, rs5128 showed a trend for association with higher TG levels in homozygous (1.10 mmol/L ± 0.15) as well as heterozygous (1.00 mmol/L ± 0.05) of the minor allele when compared to homozygous for the wild type allele (0.90 mmol/L ± 0.04).

**Table 4:** Association of the 4 APOC3 SNPs with lipid profiles in the Kuwaiti Cohort (*n* = 733)

SNP	Parameters		GG		GC		CC	*p*-value
		n		n		n		
rs5128	BMI	349	26.77 ± 0.33	131	28.03 ± 0.65	39	27.71 ± 1.05	0.08
	TC	348	4.663 ± 0.511	131	4.72 ± 0.08	39	4.58 ± 0.14	0.73
	TG	394	0.90 ± 0.04	131	1.00 ± 0.05	39	1.10 ± 0.15	0.05
	VLDL	344	0.38 ± 0.02	129	0.41 ± 0.02	38	0.48 ± 0.07	0.06
	HDL	337	1.18 ± 0.02	127	1.21 ± 0.04	36	1.19 ± 0.05	0.55
	LDL	337	3.08 ± 0.04	127	3.08 ± 0.07	36	2.92 ± 0.12	0.44
rs2070668	BMI	179	27.32 ± 0.48	251	27.32 ± 0.44	89	26.41 ± 0.61	0.42
	TC	179	4.67 ± 0.07	251	4.69 ± 0.062	88	4.64 ± 0.094	0.95
	TG	179	0.96 ± 0.06	251	0.94 ± 0.05	89	0.86 ± 0.05	0.84
	VLDL	174	0.41 ± 0.03	249	0.40 ± 0.02	88	0.36 ± 0.02	0.89
	HDL	174	1.19 ± 0.02	241	1.19 ± 0.02	85	0.17 ± 0.03	0.27
	LDL	174	3.04 ± 0.06	241	3.09 ± 0.05	85	3.07 ± 0.09	0.72
rs2854117	BMI	211	26.77 ± 0.42	235	27.38 ± 0.46	73	27.57 ± 0.74	0.50
	TC	210	4.63 ± 0.06	235	4.67 ± 0.06	73	4.79 ± 0.10	0.55
	TG	211	0.91 ± 0.05	235	0.90 ± 0.04	73	1.08 ± 0.11	0.21
	VLDL	207	0.39 ± 0.02	231	0.38 ± 0.02	73	0.46 ± 0.05	0.21
	HDL	202	1.17 ± 0.02	229	1.21 ± 0.02	69	0.18 ± 0.04	0.12
	LDL	202	3.06 ± 0.05	229	3.05 ± 0.05	69	3.14 ± 0.09	0.66
KUAPOC3N3	BMI	514	27.12 ± 0.29	5	31.59 ± 3.34	–	–	0.14
	TC	513	4.68 ± 0.04	5	4.16 ± 0.23	–	–	0.10
	TG	514	0.93 ± 0.03	5	0.80 ± 0.24	–	–	0.12
	VLDL	506	0.34 ± 0.01	5	0.36 ± 0.11	–	–	0.14
	HDL	495	1.19 ± 0.02	5	1.02 ± 0.09	–	–	0.60
	LDL	495	3.07 ± 0.04	5	2.79 ± 0.19	–	–	0.35

*SNP* Single nucleotide polymorphism, *BMI* Body mass index, *TC* Total cholesterol, *TG* Triglycerides, *VLDL* Very low-density lipoprotein, *HDL* High density lipoprotein, *LDL* Low density lipoprotein, *mmol/L* millimoles per liter

### Multivariate analysis and genetic modeling of the associated variant (rs5128)

Genetic modelling was used to test the effect of rs5128 minor allele which showed (Additional file 1: Table S5) a strong significant association (*p* = 0.02) of the dominant model in which carriers of the minor allele had an increased risk for high BMI (OR: 3.78 (1.19–11.94). In addition, carriers of rs5128 minor allele showed slightly increased levels of TG (OR: 0.89 (0.80–0.97)) and VLDL (OR: 1.11 (1.01–1.22)) based on the dominant model (*p* <  0.05). Significant association (*p* = 0.03) of rs5128 remained after multivariate analysis in which the OR value was 4.022 (CI: 1.13–14.30) for BMI implicated the minor “C” as a “risk” allele for higher BMI (Table 5).

**Table 5:** Multivariate analysis on the effect of APOC3 rs5128 on BMI, TG and VLDL in the cohort (*n* = 733) assuming a dominant genetic model

	Variable	OR	95% C.I.	*p*-value
BMI	rs5128C/G	4.02	1.13–14.28	0.03
	rs5128G/G	3.04	0.38–24.50	0.3
	Sex (Male)	1.44	0.47–4.42	> 0.001
	Age	1.14	1.09–1.19	0.52
TG	rs5128C/G	1.1	1.00–1.22	0.06
	rs5128G/G	1.17	0.99–1.38	0.07
	Sex (Male)	1.28	1.17–1.40	> 0.001
	Age	1.01	1.01–1.02	> 0.001
	BMI	1.02	1.02–1.03	> 0.001
VLDL	rs5128C/G	1.1	0.99–1.22	0.07
	rs5128G/G	1.17	0.98–1.39	0.08
	Sex (Male)	1.27	1.16–1.39	> 0.001
	Age	1.02	1.01–1.02	> 0.001
	BMI	1.02	1.02–1.03	> 0.001

*BMI* Body mass index, *TG* Triglycerides, *VLDL* Very low-density lipoprotein

## Discussion

The current study reports for the first time the full genetic profile of the *APOC3* gene locus (excluding the repetitive sequence) among Arab ethnicity (Kuwaiti Arabs) which included 42 previously reported SNPs and 3 novel variants.

Sequence and mutation analysis provided insight on the locus structure and its conservation. The ratio of substitution mutations (*n* = 43) to indels (*n* = 2) was expected to be high since InDels are subjected to strong purifying selection in order to avoid their severe functional constraint as they are more likely to disrupt protein structures or to interfere with the functions of coding, splicing, and regulatory sequence elements [28, 29]. Moreover, the rate of transition substitution was 3.3 times the rate of transversion at the *APOC3* gene locus in the studied population. Part of this transition bias is thought to be driven by underlying chemical and structural properties of DNA that favor transition mutations as they are thermodynamically more stable [30].

It has been documented that the rates of SNPs are known to vary across the functional components near genes [31, 32]. In this study, the observed SNPs were more frequent in noncoding regions (*n* = 43: intronic = 26, upstream = 12, 3′-UTR =3, 5′-UTR =2) than in the coding exons (*n* = 2), a signature of purifying selection against changes. However, this feature does not preclude a functional effect, as SNPs in noncoding sequences may have regulatory roles especially with alternative splicing [33, 34]. Low occurrence of SNPs in the coding exons (*n* = 2) could be explained by selective evolutionary pressure to maintain their structural and functional integrity [32], especially since the studied population were apparently healthy at the time of data collection. However, low variability occurs not only in protein-coding regions but also in non-coding regions harboring exons including both 5′-UTR (*n* = 2) and 3′-UTR (*n* = 3), this accentuates the importance of conservation among such regions because of their sequence-dependent role in gene regulation through mRNA processing and translation [32–35].

Overabundance of SNPs in noncoding regions, especially introns (*n* = 26), could be explained simply by the evolution pressure in regions with less genomic sequence conservation being relatively low compared to regions encoding sequence-dependent functions [32]. However, this hypothesis cannot explain the observed polymorphisms accumulation in the promoter region, wherein 12 SNPs including a novel variant were detected in our study. One possible explanation is that these variants were introduced with some functional importance or role throughout the human evolutionary history in which *APOC3* gene was under continuous natural selection pressure and alteration by mutation, genetic drift, and gene flow [36].

The allele frequency distribution at the *APOC3* loci was compared to other reported populations. The overall pattern of allelic frequencies of *APOC3* common SNPs (MAF higher than 5%) in the sampled population of Kuwaiti Arabs (*n* = 100) were found to be fairly comparable to the frequencies of other populations obtained from the 1000GENOMES and HapMap deposited in ensembl including Caucasion, American, Asian, and European (Additional file 1: Table S3) [37]. However, some SNPs showed large interethnic variations in their allelic frequencies. The largest ethnic variation in the allelic frequencies of the common *APOC3* SNPs (MAF higher than 5%) was observed when compared to African population [37].

Most polymorphisms in *APOC3* gene promoter are thought to play some role in the regulation of APOC3 expression. Therefore, the evaluation of the allelic distribution of some common promoter variations (rs2542052, rs10892037, rs11568823, rs2854116, and rs2854117) in various ethnic groups is crucial in understanding the interethnic variability in *APOC3* activity. The common SNPs at sites A-641C (rs2542052), A-630G (rs10892037), −625insT (rs11568823) of the promoter region are representative to each other. Kuwaiti Arabs (*n* = 100) showed higher frequencies (49.5%) for the previously reported “variant” promoter alleles at sites -641A, −630A, and -625del when compared to Caucasions (42%) [15, 16]. In regard to the two common SNPs, T-455C (rs2854116) and C-482 T(rs2854117) observed within the insulin responsive element (IRE) in the promoter region, the allelic frequencies differed markedly among ethnic groups. The frequency of the -482C allele (rs2854117) detected in Kuwaiti Arabs (37%) is lower than that of Chinese Han population (53.45%) [4]. Kozlitina and his colleagues investigated the frequency of the same polymorphism in different ethnic groups and reported the highest frequency in African American (71.2%) and less common in Hispanics (38.9%) and Europeans (36.6%). These interethnic differences in the allele frequencies of the two common variatiants within IRE suggest that there may be potential ethnic differences in *APOC3* expression downregulation pathway activity and IRE sensitivity [26]. The frequency of the common variant rs5128 (C3238G), in the 3′-UTR was also found to be different from other reports. A higher frequency of the rare 3238G allele (19.6%) was observed in Kuwaiti Arabs than Caucasians (0–11%), comparable to the frequencies reported for Saudi Arabians (18%), Iranians (14%) and Costa Ricans (19%) [17, 25, 38–41]; while being lower than those reported for Northwest Indian subpopulations (22.6–26.2%) Asian Indians (31.3%) [42, 43].

The haplotype structure exhibited a complete LD (*r*^2^ = 1) observed between 4 promoter SNPs, rs2542052 (A-641C), rs10892037 (A-630G), rs11568823 (− 625insT) and rs2854116 (T-455C), all of which were within 186 base pairs. Considering the polymorphism at site − 455 (rs2854116), no previous studies (to our knowledge) have shown complete LD with the 3 concordant promoter SNPs listed above. Dammerman study [15] on Caucasians showed a strong LD rather than a complete LD in which the − 625 (rs11568823) genotype predicted the − 455 (rs2854116) genotype in 169 out of 173 subjects [43]. Moreover, Brown and his colleagues also generated a very strong LD in the place of complete LD within this polymorphisms pair (rs11568823 and rs2854116) [17].

In the present study, 2 additional haplotypes blocks were observed beside the common promoter haplotype reported in other studies. Both of them are three-marker haplotypes found within moderate disequilibrium regions. The first of the two encompasses 3 sequential SNPs within the first intron (rs734104, rs2070669, and rs2070668) while the second block covers rs5130 in intron 3 along with both rs5128 and rs4225 within the untranslated region of exon 4. The emergence of both haplotypes in Kuwaiti Arabs population could be explained in view of population-level behavior of alleles at adjacent loci or interethnic allele frequency differences. The aggregation of the above-mentioned SNPs in each haplotype suggest that each 3 variants may functionally cooperate in the Kuwaiti Arabs population. Based on these findings, the variants analyzed for association in this study were selected according to their haplotype group.

Studies of the association between various *APOC3* polymorphisms and lipid profile have reported apparently conflicting findings across different populations [18, 43–46]. In the studied cohort (*n* = 733) and among the 5 tested *APOC3* SNPs (rs5128, rs2854117, rs2070668, and Novels SNP 2 and 3), only rs5128 showed an association with increased BMI, TG and VLDL levels (Table 4) that was further validated using multivariate analysis. The rs5128 variant was found to be significantly associated (*p* <  0.05) with BMI among the studied cohort following a dominant genetic model (Table 5) and increased risk by an OR of 4.022 (CI: 1.13–14.30). Such an association was in concordance with other studies reported in different population [18–27]. It must be noted that the deviation from HWE observed for rs5128 was most likely an outcome of excessive homozygous carriers of the mutant wild type C allele conferring to the above findings and is not an outcome of genotyping error. Randomly selected samples were genotyped, and the results were consistent. In addition, other studied variants such as rs2070668 conferred to HWE further supporting the potential involvement of rs5128 with BMI in a known population prevalent with obesity.

The contributed small effect of the rs5128 minor allele, localized in the 3″UTR of exon 4, could be the outcome of possible increased transcriptional activity of the gene resulting in higher plasma levels of APOC3 protein. Studies have reported that high APOC3 levels is directly correlated to increased levels of TG, VLDL, and BMI [47]. There are three different possible mechanisms involved in the elevation of TG levels by APOC3. First, APOC3 is an inhibitor of lipoprotein lipase (LPL), a key rate limiting enzyme in the hydrolysis of TG-rich particles thereby increased levels of APOC3 would increases the inhibition of LPL [47]. Second, APOC3 promotes the assembly and secretion of VLDL in the liver yielding higher circulation of VLDL in the bloodstream [48, 49]. Thirdly, at higher concentrations APOC3, inhibition of hepatic lipase (HL) activity may also occur leading to a delayed catabolism of TG-rich particles [50]. Other indirect mechanisms by which the APOC3 could affect lipid metabolism resulting in accumulation of TG have been suggested [51]. A more recent study reported that another variant (rs4225) in the vicinity had a role in the regulation of gene expression and increased TG levels through the introduction of an miR-4271 binding site [21]. Furthermore, it could be postulated that such variants could have a modified affect related to nutraceuticals in regulating lipid levels. These molecules as well as functional food ingredients have been shown [52] to affect lipid levels and more specifically may reduce VLDL levels and that the mechanism behind this could be under genetic control.

## Conclusion

*APOC3* was found to be highly polymorphic in the studied Kuwaiti Arab population in which 42 previously reported SNPs and 3 novel SNPs were identified, one of which as characterized as “very rare” variant which may make a useful maker for ethnic identification. Only rs5128 showed an association with increased BMI, TG and VLDL levels in which the G allele is a risk variant and was found to deviate from HWE most likely as a result of its association and not the outcome of sampling error. This study supports the inclusion of rs5128 a marker for assessing genetic risk to dyslipidemia. For future studies and considering the importance of the repetitive sequences in genetic control processes, it would be interesting to analyze the repetitive sequence of the *APOC3* gene among different ethnic groups employing a specific and reproducible genotyping protocol. Repeats have always presented technical challenges for sequence alignment and assembly programs. Polymorphisms of the repetitive sequence may be genotyped by targeted PCR with primers flanking the repetitive sequence and examining the resolving the products on high resolution gels that would facilitate the identification of the repeat alleles. The limitation of this study is in the lack of apolipoprotein and LPL levels.

## Supplementary information

**Additional file 1: Table S1.** Primers sets designed to sequence the full APOC3 gene. **Table S2.** PCR thermal profile used for all the primer sets of the APOC3 gene in this study during both amplification and sequencing reactions. **Table S3.** A summary of genotypic and allelic frequencies for the APOC3 SNPs showing within the total population (*n* = 100). Listed are the number of the SNP when used in the linkage disequilibrium and haplotype analysis (first column), the dbSNP reference number. **Table S4.** Pairwise test of linkage-disequilibrium as measured by r2 between the identified 22 segregating SNPs at the APOC3 gene locus (MAF > 5%). **Table S5.** Genetic modeling of APOC3 rs5128 with BMI, TG, and VLDL. **Table S6.** Minor allelic frequencies of commonly studied APOC3 SNPs (MAF > 5%) in reported in various populations.

**Additional file 2: Figure S1.** Amplification plots for the genotypes of the identified novel APOC3 variants as observed by real-time PCR.

## Data Availability

Additional data and analysis are provided in the supplementary files. Any other data may be made available upon request from the corresponding author.

## Abbreviations

3′-UTR: 3′-untranslated region
5′-UTR: 5′-untranslated region
*APOC3*: Apolipoprotein C3 human gene
APOC3: Apolipoprotein C3 human protein
BMI: Body mass index
bp: Base pair
dbSNP: The single nucleotide polymorphisms database
DNA: Deoxyribonucleic acid
GMAF: Global minor allele frequency
HDL-C: High-density-lipoprotein-cholesterol
HWE: Hardy-Weinberg equilibrium
Indel: Insertion or deletion
Kb: Kilo base
LDL-C: Low-density lipoprotein-cholesterol
NCBI: National center for biotechnology information
PCR: Polymerase chain reaction
rs: Reference sequence
SNPs: Single nucleotide polymorphisms
TC: Total cholesterol
TG: Triglycerides
