# Supplementary material for: Sequence analysis and variant identification at the APOC3 gene locus indicates association of rs5218 with BMI in a sample of Kuwaiti’s
Source: Lipids Health Dis. 2019 Dec 19;18:224. doi: 10.1186/s12944-019-1165-6 (PMC6921598; doi:10.1186/s12944-019-1165-6)
Supplement: Supplementary file 1 — Additional file 1: Table S1. Primers sets designed to sequence the full APOC3 gene. Table S2. PCR thermal profile used for all the primer sets of the APOC3 gene in this study during both amplification and sequencing reactions. Table S3. A summary of genotypic and allelic frequencies for the APOC3 SNPs showing within the total population (n = 100). Listed are the number of the SNP when used in the linkage disequilibrium and haplotype analysis (first column), the dbSNP reference number. Table S4. Pairwise test of linkage-disequilibrium as measured by r2 between the identified 22 segregating SNPs at the APOC3 gene locus (MAF > 5%). Table S5. Genetic modeling of APOC3 rs5128 with BMI, TG, and VLDL. Table S6. Minor allelic frequencies of commonly studied APOC3 SNPs (MAF > 5%) in reported in various populations. [file 12944_2019_1165_MOESM1_ESM.docx]

**Supplementary Tables**

**Table S1:** Primers sets designed to sequence the full APOC3 gene.

| **Primer set** | **F/R** | **Sequence (5' to 3')** | **n moles** | **µl for 100 µM *** | **Tm °C** | **bp** |
| --- | --- | --- | --- | --- | --- | --- |
| ***APOC3* - 1** | F | GGGTCCTATTACGTGCCAAA | 36.1 | 361 | 58.36 | 569 |
|  | R | CCTCCATCTCTGGGTTTCAA | 40.4 | 404 | 58.5 |  |
| ***APOC3* -2** | F | CAACCCCTGCCCTACACTAA | 30.2 | 302 | 58.5 | 575 |
|  | R | CTGCTGACCAGTGGAGATGA | 31.5 | 315 | 58.32 |  |
| ***APOC3* -3** | F | CAGCGTGGACTCAGTCTCCT | 40.03 | 403 | 59.00 | 526 |
|  | R | CAGTTTCCCTGTCTGGGGTA | 48.1 | 481 | 58.43 |  |
| ***APOC3* -4** | F | GCTGGAAGTGGCTCCAAGT | 38.5 | 385 | 58.83 | 558 |
|  | R | CTTGACCACCCATTGGGACT | 44 | 440 | 60.58 |  |
| ***APOC3* -5** | F | TTCCTTGCAGGAACAGAGGT | 24.6 | 246 | 58.3 | 538 |
|  | R | GGGAGGAATGCAGTCTCAAC | 29.7 | 297 | 58.1 |  |
| ***APOC3* -6** | F | GCCTCTTCAGCCTCCTCTTT | 31.7 | 317 | 58.62 | 687 |
|  | R | TGAGGACCACTGCTCTAGCC | 30.1 | 301 | 58.99 |  |
| ***APOC3* -7** | F | CCCATTTTCCTTTTCTGAAGG | 36.8 | 368 | 58.52 | 537 |
|  | R | GTCAGGGTGAGGTGGAGAAG | 37.7 | 377 | 58.09 |  |
| ***APOC3* -8** | F | TGTGTCCTTCCTCTCCCATC | 27.8 | 278 | 58.45 | 540 |
|  | R | CCACACCACCCTCTCAACTT | 31.8 | 318 | 58.43 |  |
| ***APOC3* -9** | F | GATGGCAGGGTTTGACTTGT | 30.9 | 309 | 58.43 | 569 |
|  | R | AAGCCTGTGCCACTACATCC | 19.1 | 191 | 58.08 |  |

Listed are the nucleotide sequence of each primer, the amount of nmoles, volume of ddH2O required to prepare 100 µM stock primer, melting temperature (Tm), and the product size of each primers set.

**Table S2:** PCR thermal profile used for all the primer sets of the APOC3 gene in this study during both amplification and sequencing reactions.

| **Parameter** | **Temperature (°C)** | **Time** | **Number of Cycles** |
| --- | --- | --- | --- |
| **Amplification PCR** | | | |
| **Initial denaturation** | 94 | 5 minutes | 1 |
| **Denaturation** | 94 | 30 seconds | 35 |
| **Annealing** | 58 | 30 seconds |  |
| **Extension** | 72 | 30 seconds |  |
| **Final Extension** | 72 | 7 minutes | 1 |
| **Hold** | 4 | - | - |
| **Sequencing PCR** | | | |
| **Initial Denaturation** | 96°C | 1 minute | 1 |
| **Denaturation** | 96°C | 10 seconds | 25 |
| **Annealing** | 50°C | 5 seconds |  |
| **Extension** | 60°C | 1:15 minutes |  |

**Table S3:** A summary of genotypic and allelic frequencies for the APOC3 SNPs showing within the total population (n=100). Listed are the number of the SNP when used in the linkage disequilibrium and haplotype analysis (first column), the dbSNP reference number.

| **Sl. No.** | **SNPs** | **Location on gene** (NG_008949.1) | **Genotypic Frequencies** | | | **Allelic Frequencies** | | ***P-value for HWE** |
| --- | --- | --- | --- | --- | --- | --- | --- | --- |
|  |  |  | **n = 100** | | |  |  |  |
| **SNPs showing MAF >5%** | | | | | | | | |
| 1 | rs12721080 | g.4058G>A | GG= 61% | GA= 36% | AA= 3% | G= 79% | A= 21% | 0.395 |
| 2 | rs2542052 | g.4361A>C | AA= 25% | AC=49% | CC=26% | A= 49.5% | C= 50.5% | 0.842 |
| 3 | rs10892037 | g.4372A>G | AA= 25% | AG=49% | GG=26% | A= 49.5% | G= 50.5% | 0.842 |
| 4 | rs11568823 | g.4376_4377insT | --= 25% | -T=49% | TT=26% | -= 49.5% | T= 50.5% | 0.842 |
| 5 | rs2854117 | g.4519T>C | TT=57% | TC=7% | CC=36% | T= 60.5% | C= 39.5% | **<0.001** |
| 6 | rs2854116 | g.4546C>T | CC= 25% | CT= 49% | TT=26% | C=49.5% | T=50.5% | 0.842 |
| 7 | rs618354 | g.5154G>C | GG=51% | GC=40% | CC=9% | G=55.5% | C= 44.5% | 0.774 |
| 8 | rs734104 | g.5237C>T | CC= 8% | CT= 24% | TT=68% | T= 80% | C= 20% | 0.012 |
| 9 | rs2070669 | g.5499C>G | CC=19% | CG=50% | GG=31% | G= 56% | C= 44% | 0.883 |
| 10 | rs2070668 | g.5530T>G | TT= 39% | TG= 44% | GG= 17% | T= 61% | G= 39% | 0.452 |
| 11 | rs4520 | g.5912T>C | TT=16% | TC=44% | CC=40% | T= 38% | C= 62% | 0.508 |
| 12 | rs2070667 | g.6046G>A | GG= 87% | GA= 13% | AA=0% | G= 93.5% | A= 6.5% | 0.487 |
| 13 | rs2070666 | g.6051T>A | TT=67% | TA=28% | AA=5% | T= 81% | A= 19% | 0.366 |
| 14 | rs1269330 | g.6210G>A | GG= 89% | GA= 11% | AA=0% | G= 94.5% | A= 5.5% | 0.561 |
| 15 | rs5142 | g.6227T>C | TT=10% | TC=21% | CC=69% | T= 20.5 | C= 79.5 | **0.000375** |
| 16 | rs5141 | g.6500T>C | CC=77% | TC=17% | TT=6% | C= 85.5% | T= 14.5% | **0.001668** |
| 17 | rs553080 | g.6722T>C | TT=87% | TC=12% | CC=1% | T= 93% | C= 7% | 0.433 |
| 18 | rs645901 | g.6722T>C | TT=10% | TC=27% | CC=63% | T= 23.5 | C= 76.5% | 0.013 |
| 19 | rs5134 | g.6956T>C | TT=88% | CT=12% | CC=0% | T= 94% | C= 6% | 0.523 |
| 20 | rs5130 | g.7523C>T | CC=9% | CT=30% | TT=61% | C= 24% | T= 76% | 0.076 |
| 21 | rs5128 | g.8017G>C | GG=7% | GC=21% | CC=72% | G= 17.5% | C= 82.5% | 0.006 |
| 22 | rs4225 | g.8048G>T | GG=29% | GT=50% | TT=21% | G= 54% | T= 46% | 0.949 |
| **SNPs showing MAF <5%** | | | | | | | | |
| 23 | rs12721079 | g.4025C>T | CC= 92% | CT = 8% | TT = 0% | C= 96% | T= 4% | 0.677 |
| 24 | rs12721077 | g.4057C>T | CC= 98% | CT= 2% | TT= 0% | C= 99% | T= 1% | 0.919 |
| 25 | rs12721081 | g.4171C>T | CC= 98% | CT= 2% | TT = 0% | C= 99% | T= 1% | 0.919 |
| 26 | rs113643578 | g.4184C>A | CC= 98% | CA= 2% | AA= 0% | C= 99% | A= 1% | 0.919 |
| 27 | rs145834983 | g.4285G>A | GG= 99% | GA= 1% | AA= 0% | G= 99.5% | A= 0.5% | 0.960 |
| 28 | NOVEL 1 | g.4976_4977insA | --= 98% | -A= 2 | AA= 0% | -= 99% | A= 1% | 0.919 |
| 29 | rs191196015 | g.5064G>A | GG= 96% | GA=4% | AA= 0% | G= 98% | A= 2% | 0.838 |
| 30 | rs12721090 | g.5152C>T | CC= 98% | CT= 2% | TT = 0% | C= 99% | T= 1% | 0.919 |
| 31 | rs11827682 | g.5162C>T | CC= 96% | CT=4% | TT= 0% | C= 98% | T= 2% | 0.838 |
| 32 | NOVEL 2 | g.5196 A>G | AA= 99% | AG= 1% | GG= 0% | A= 99.5% | G= 0.5% | 0.960 |
| 33 | NOVEL 3 | g. 5536G>A | GG= 99% | GA= 1% | AA=0% | G= 99.5% | A= 0.5% | 0.960 |
| 34 | rs192830070 | g.5581C>T | CC=99% | CT=1% | TT=0% | C= 99.5% | T= 0.5% | 0.960 |
| 35 | rs5143 | g.5593G>A | GG= 99% | GA= 1% | AA=0% | G= 99.5% | A= 0.5% | 0.960 |
| 36 | rs12721095 | g.5601C>T | CC=97% | CT=3% | TT=0% | C= 98.5% | T= 1.5% | 0.879 |
| 37 | rs76353203 | g.5730C>T | CC= 99% | CT=1% | TT=0% | C= 99.5% | T= 0.5% | 0.960 |
| 38 | rs145735257 | g.6231G>A | GG=99~~%~~ | GA=1% | AA=0% | G= 99.5% | A= 0.5% | 0.960 |
| 39 | rs184842636 | g.6498C>T | CC=99% | CT=1% | TT=0% | C= 99.5% | T= 0.5% | 0.960 |
| 40 | rs5140 | g.6544G>A | GG=99% | GA=1% | AA=0% | G= 99.5% | A= 0.5% | 0.960 |
| 41 | rs12721098 | g.6606C>T | CC=99% | CT=1% | TT=0% | C= 99.5% | T= 0.5% | 0.960 |
| 42 | rs5132 | g.7155C>T | CC=91% | CT=9% | TT=0% | C= 95.5% | T= 4.5% | 0.637 |
| 43 | rs12721084 | g.7775G>A | GG=99% | GA=1% | AA=0% | G= 99.5% | A= 0.5% | 0.960 |
| 44 | rs111429645 | g.7780G>T | GG=98% | GT=2% | TT=0% | G= 99% | T= 1% | 0.919 |
| 45 | rs187628630 | g.8116C>G | CC=98% | CG=2% | GG=0% | C= 99% | G= 1% | 0.919 |

*P-values are generated by chi-square test.

* **Values in bold** indicate significant deviation from HWE after Bonferroni correction (p-value <0.00227).

| **SNPs** | | **1** | **2** | **3** | **4** | **5** | **6** | **7** | **8** | **9** | **10** | **11** | **12** | **13** | **14** | **15** | **16** | **17** | **18** | **19** | **20** | **21** | **22** |
| --- | --- | --- | --- | --- | --- | --- | --- | --- | --- | --- | --- | --- | --- | --- | --- | --- | --- | --- | --- | --- | --- | --- | --- |
|  |  | **rs12721080** | **rs2542052** | **rs10892037** | **rs11568823** | **rs2854117** | **rs2854116** | **rs618354** | **rs734104** | **rs2070669** | **rs2070668** | **rs4520** | **rs2070667** | **rs2070666** | **rs1269330** | **rs5142** | **rs5141** | **rs553080** | **rs645901** | **rs5134** | **rs5130** | **rs5128** | **rs4225** |
| **1** | **rs12721080** | 1 | 0.271 | 0.271 | 0.271 | 0.002 | 0.271 | 0.651 | 0.066 | 0.023 | 0.17 | 0.128 | 0.018 | 0.007 | 0.015 | 0.029 | 0.045 | 0.02 | 0.082 | 0.017 | 0.084 | 0.056 | 0.272 |
| **2** | **rs2542052** | 0.271 | 1 | 1 | 1 | 0.169 | 1 | 0.38 | 0.166 | 0.581 | 0.555 | 0.072 | 0.024 | 0.004 | 0.018 | 0.107 | 0.173 | 0.011 | 0.117 | 0.021 | 0.197 | 0.134 | 0.034 |
| **3** | **rs10892037** | 0.271 | 1 | 1 | 1 | 0.169 | 1 | 0.38 | 0.166 | 0.581 | 0.555 | 0.072 | 0.024 | 0.004 | 0.018 | 0.107 | 0.173 | 0.011 | 0.117 | 0.021 | 0.197 | 0.134 | 0.034 |
| **4** | **rs11568823** | 0.271 | 1 | 1 | 1 | 0.169 | 1 | 0.38 | 0.166 | 0.581 | 0.555 | 0.072 | 0.024 | 0.004 | 0.018 | 0.107 | 0.173 | 0.011 | 0.117 | 0.021 | 0.197 | 0.134 | 0.034 |
| **5** | **rs2854117** | 0.002 | 0.169 | 0.169 | 0.169 | 1 | 0.169 | 0.012 | 0.121 | 0.276 | 0.101 | 0.084 | 0.015 | 0.001 | 0.009 | 0.062 | 0.111 | 0.008 | 0.051 | 0.003 | 0.092 | 0.098 | 0.064 |
| **6** | **rs2854116** | 0.271 | 1 | 1 | 1 | 0.169 | 1 | 0.38 | 0.166 | 0.581 | 0.555 | 0.072 | 0.024 | 0.004 | 0.018 | 0.107 | 0.173 | 0.011 | 0.117 | 0.021 | 0.197 | 0.134 | 0.034 |
| **7** | **rs618354** | 0.651 | 0.38 | 0.38 | 0.38 | 0.012 | 0.38 | 1 | 0.102 | 0.097 | 0.261 | 0.046 | 0.003 | 0.021 | 0.001 | 0.063 | 0.069 | 0.002 | 0.097 | 0 | 0.097 | 0.087 | 0.078 |
| **8** | **rs734104** | 0.066 | 0.166 | 0.166 | 0.166 | 0.121 | 0.166 | 0.102 | 1 | 0.318 | 0.16 | 0.299 | 0.009 | 0.036 | 0.015 | 0.683 | 0.678 | 0.019 | 0.428 | 0.016 | 0.589 | 0.848 | 0.156 |
| **9** | **rs2070669** | 0.023 | 0.581 | 0.581 | 0.581 | 0.276 | 0.581 | 0.097 | 0.318 | 1 | 0.434 | 0.153 | 0.019 | 0.001 | 0.007 | 0.174 | 0.216 | 0.001 | 0.199 | 0.001 | 0.299 | 0.27 | 0.132 |
| **10** | **rs2070668** | 0.17 | 0.555 | 0.555 | 0.555 | 0.101 | 0.555 | 0.261 | 0.16 | 0.434 | 1 | 0.016 | 0.001 | 0.05 | 0.004 | 0.113 | 0.108 | 0.048 | 0.108 | 0.004 | 0.202 | 0.136 | 0.116 |
| **11** | **rs4520** | 0.128 | 0.072 | 0.072 | 0.072 | 0.084 | 0.072 | 0.046 | 0.299 | 0.153 | 0.016 | 1 | 0.003 | 0.383 | 0 | 0.216 | 0.24 | 0.007 | 0.17 | 0 | 0.268 | 0.309 | 0.453 |
| **12** | **rs2070667** | 0.018 | 0.024 | 0.024 | 0.024 | 0.015 | 0.024 | 0.003 | 0.009 | 0.019 | 0.001 | 0.003 | 1 | 0.005 | 0.68 | 0.002 | 0.012 | 0.639 | 0.127 | 0.76 | 0.168 | 0.015 | 0.059 |
| **13** | **rs2070666** | 0.007 | 0.004 | 0.004 | 0.004 | 0.001 | 0.004 | 0.021 | 0.036 | 0.001 | 0.05 | 0.383 | 0.005 | 1 | 0.009 | 0.042 | 0.04 | 0.018 | 0.072 | 0.015 | 0.074 | 0.05 | 0.2 |
| **14** | **rs1269330** | 0.015 | 0.018 | 0.018 | 0.018 | 0.009 | 0.018 | 0.001 | 0.015 | 0.007 | 0.004 | 0 | 0.68 | 0.009 | 1 | 0 | 0.01 | 0.773 | 0.138 | 0.742 | 0.133 | 0.012 | 0.023 |
| **15** | **rs5142** | 0.029 | 0.107 | 0.107 | 0.107 | 0.062 | 0.107 | 0.063 | 0.683 | 0.174 | 0.113 | 0.216 | 0.002 | 0.042 | 0 | 1 | 0.658 | 0.003 | 0.45 | 0.001 | 0.52 | 0.763 | 0.116 |
| **16** | **rs5141** | 0.045 | 0.173 | 0.173 | 0.173 | 0.111 | 0.173 | 0.069 | 0.678 | 0.216 | 0.108 | 0.24 | 0.012 | 0.04 | 0.01 | 0.658 | 1 | 0.013 | 0.451 | 0.011 | 0.486 | 0.732 | 0.115 |
| **17** | **rs553080** | 0.02 | 0.011 | 0.011 | 0.011 | 0.008 | 0.011 | 0.002 | 0.019 | 0.001 | 0.048 | 0.007 | 0.639 | 0.018 | 0.773 | 0.003 | 0.013 | 1 | 0.147 | 0.848 | 0.142 | 0.016 | 0.036 |
| **18** | **rs645901** | 0.082 | 0.117 | 0.117 | 0.117 | 0.051 | 0.117 | 0.097 | 0.428 | 0.199 | 0.108 | 0.17 | 0.127 | 0.072 | 0.138 | 0.45 | 0.451 | 0.147 | 1 | 0.208 | 0.763 | 0.49 | 0.173 |
| **19** | **rs5134** | 0.017 | 0.021 | 0.021 | 0.021 | 0.003 | 0.021 | 0 | 0.016 | 0.001 | 0.004 | 0 | 0.76 | 0.015 | 0.742 | 0.001 | 0.011 | 0.848 | 0.208 | 1 | 0.202 | 0.014 | 0.054 |
| **20** | **rs5130** | 0.084 | 0.197 | 0.197 | 0.197 | 0.092 | 0.197 | 0.097 | 0.589 | 0.299 | 0.202 | 0.268 | 0.168 | 0.074 | 0.133 | 0.52 | 0.486 | 0.142 | 0.763 | 0.202 | 1 | 0.672 | 0.238 |
| **21** | **rs5128** | 0.056 | 0.134 | 0.134 | 0.134 | 0.098 | 0.134 | 0.087 | 0.848 | 0.27 | 0.136 | 0.309 | 0.015 | 0.05 | 0.012 | 0.763 | 0.732 | 0.016 | 0.49 | 0.014 | 0.672 | 1 | 0.152 |
| **22** | **rs4225** | 0.272 | 0.034 | 0.034 | 0.034 | 0.064 | 0.034 | 0.078 | 0.156 | 0.132 | 0.116 | 0.453 | 0.059 | 0.2 | 0.023 | 0.116 | 0.115 | 0.036 | 0.173 | 0.054 | 0.238 | 0.152 | 1 |

**Table S4:** Pairwise test of linkage-disequilibrium as measured by r2 between the identified 22 segregating SNPs at the APOC3 gene locus (MAF>5%).

* A value of 1 indicates complete linkage disequilibrium.

**Table S5:** Genetic modeling of APOC3 rs5128 with BMI, TG, and VLDL.

| Variant association | Model | Genotypes | n | OR | p-value |
| --- | --- | --- | --- | --- | --- |
| rs5128 with BMI | Codominant | C/C | 349 (67%) | 1 | 0.15 |
|  |  | C/G | 131 (25%) | 4.02( 1.13 - 14.28 ) |  |
|  |  | G/G | 39 (8%) | 3.03 ( 0.38 - 24.50 ) |  |
|  | Dominant | C/C | 349 (67%) | 1 | **0.02** |
|  |  | C/G-G/G | 170 (33%) | 3.77 ( 1.19 - 11.98 ) |  |
|  | Recessive | C/C-C/G | 480 (92%) | 1 | 0.49 |
|  |  | G/G | 39 (8%) | 2.08 ( 0.26 - 16.36 ) |  |
|  | Overdominant | C/C-G/G | 388 (75%) | 1 | 0.05 |
|  |  | C/G | 131 (25%) | 3.60 ( 1.03 - 12.54 ) |  |
|  | log-Additive | 0,1,2 |  | 2.39 ( 1.01 - 5.74 ) | 0.05 |
| rs5128 with TG | Codominant | C/C | 347 (69%) | 1 | **0.05** |
|  |  | C/G | 140 (27%) | 1.10 ( 1.00 - 1.22 ) |  |
|  |  | G/G | 19 (4%) | 1.17 ( 0.99 - 1.38 ) |  |
|  | Dominant | C/C | 347 (69%) | 1 | **0.02** |
|  |  | C/G-G/G | 159 (31%) | 1.12 ( 1.02 - 1.23 ) |  |
|  | Recessive | C/C-C/G | 487 (96%) | 1 | **0.01** |
|  |  | G/G | 19 (4%) | 1.13 ( 0.96 - 1.34 ) |  |
|  | Overdominant | C/C-T/T | 366 (72%) | 1 | 0.1 |
|  |  | C/T | 140 (28%) | 1.09 ( 0.98 - 1.20 ) |  |
|  | log-Additive | 0,1,2 |  | 1.09 ( 1.02 - 1.17 ) | 0.01 |
| rs5128 with VLDL | Codominant | C/C | 344 (67%) | 1 | 0.07 |
|  |  | C/G | 129 (25%) | 1.10 ( 0.99 - 1.22 ) |  |
|  |  | G/G | 38 (8%) | 1.17 ( 0.98 - 1.39 ) |  |
|  | Dominant | C/C | 344 (67%) | 1 | **0.03** |
|  |  | C/G-G/G | 167 (33%) | 1.11 ( 1.01 - 1.23) |  |
|  | Recessive | C/C-C/G | 473 (92%) | 1 | 0.13 |
|  |  | G/G | 38 (8%) | 1.14 ( 0.96 - 1.35 ) |  |
|  | Overdominant | C/C-G/G | 382 (75%) | 1 | 0.13 |
|  |  | C/G | 129 (25%) | 1.08 ( 0.98 - 1.20 ) |  |
|  | log-Additive | 0,1,2 |  | 1.09 ( 1.01 - 1.17 ) | **0.02** |

**Table S6: Minor allelic frequencies of commonly studied APOC3 SNPs (MAF > 5%) in reported in various populations.**

| SNPs | | Kuwaiti Arabs | Caucasian | African | American | Asian | European | GMAF |  |  |
| --- | --- | --- | --- | --- | --- | --- | --- | --- | --- | --- |
|  |  | References | | | | | | | | |
|  |  | Present  Study* | 1000 Genomes Project [36] | | | | | | |  |
| 1 | rs2542052 | A: 49.5% | A: 38.8% | C: 27% | A: 47% | A: 50% | A: 42% | C: 48% |  |  |
| 2 | rs10892037 | A: 49.5% | A: 38.8% | G: 25% | A: 49% | A: 49% | A: 42% | G: 48% |  |  |
| 3 | rs11568823 | -: 49.5% | -:0.37.1% | T: 26% | -: -: 47% | -: -: 48% | -: -: 40% | T:49% |  |  |
| 4 | rs2854117 | C: 37% | T: 28.2% | C: 26% | T: 36% | T: 47% | T: 30% | T:46% |  |  |
| 5 | rs2854116 | T: 38% | C: 37.6% | T: 24% | C: 44% | C: 48% | C: 39% | T: 49% |  |  |
| 6 | rs2070668 | G: 39% | T: 43.5% | G: 13% | G: 48% | G: 40% | T: 45% | G: 40% |  |  |
| 7 | rs5128 | G: 19.6% | G: 10.6% | G: 14% | G: 16% | G: 35% | G: 12% | G :19% |  |  |

SNP, Single nucleotide polymorphism; GMAF, Global minor allele frequency;

*The frequencies for SNPs 1-3 are those estimated from the initial Kuwaiti Arab samples sequenced samples and SNPS 4-7 are estimated from the cohort.
