# Supplementary figures and images for: Sequence analysis and variant identification at the APOC3 gene locus indicates association of rs5218 with BMI in a sample of Kuwaiti’s
Source: Lipids Health Dis. 2019 Dec 19;18:224. doi: 10.1186/s12944-019-1165-6 (PMC6921598; doi:10.1186/s12944-019-1165-6)

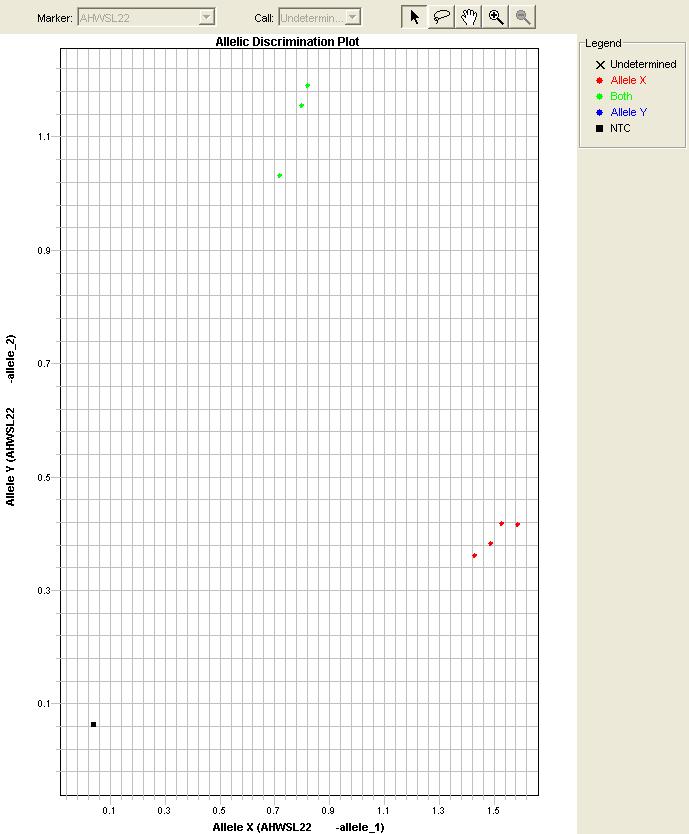

Supplement: Supplementary file 2 — Additional file 2: Figure S1. Amplification plots for the genotypes of the identified novel APOC3 variants as observed by real-time PCR. [file 12944_2019_1165_MOESM2_ESM.jpg]
